# Estimating mangrove forest gross primary production by quantifying environmental stressors in the coastal area

**DOI:** 10.1038/s41598-022-06231-6

**Published:** 2022-02-09

**Authors:** Yuhan Zheng, Wataru Takeuchi

**Affiliations:** Institute of Industrial Science, The University of Tokyo, Tokyo, 1538505 Japan

**Keywords:** Photosynthesis, Ecological modelling, Forestry, Wetlands ecology

## Abstract

Mangrove ecosystems play an important role in global carbon budget, however, the quantitative relationships between environmental drivers and productivity in these forests remain poorly understood. This study presented a remote sensing (RS)-based productivity model to estimate the light use efficiency (LUE) and gross primary production (GPP) of mangrove forests in China. Firstly, LUE model considered the effects of tidal inundation and therefore involved sea surface temperature (SST) and salinity as environmental scalars. Secondly, the downscaling effect of photosynthetic active radiation (PAR) on the mangrove LUE was quantified according to different PAR values. Thirdly, the maximum LUE varied with temperature and was therefore determined based on the response of daytime net ecosystem exchange and PAR at different temperatures. Lastly, GPP was estimated by combining the LUE model with the fraction of absorbed photosynthetically active radiation from Sentinel-2 images. The results showed that the LUE model developed for mangrove forests has higher overall accuracy (RMSE = 0.0051, R^2^ = 0.64) than the terrestrial model (RMSE = 0.0220, R^2^ = 0.24). The main environmental stressor for the photosynthesis of mangrove forests in China was PAR. The estimated GPP was, in general, in agreement with the in-situ measurement from the two carbon flux towers. Compared to the MODIS GPP product, the derived GPP had higher accuracy, with RMSE improving from 39.09 to 19.05 g C/m^2^/8 days in 2012, and from 33.76 to 19.51 g C/m^2^/8 days in 2015. The spatiotemporal distributions of the mangrove GPP revealed that GPP was most strongly controlled by environmental conditions, especially temperature and PAR, as well as the distribution of mangroves. These results demonstrate the potential of the RS-based productivity model for scaling up GPP in mangrove forests, a key to explore the carbon cycle of mangrove ecosystems at national and global scales.

## Introduction

Mangrove forest is one of the most carbon-rich ecosystems whose carbon sequestration is considerably higher than terrestrial forests^1^. The estimate of the gross primary production (GPP) is important to understand the carbon cycle in mangrove ecosystems. Carbon flux data measured with eddy covariance (EC) techniques provide invaluable information on ecosystem productivities and can be used to establish productivity models^2^. However, these models were limited to a 0.1–2 km spatial footprint around the towers, and therefore, applying them at other sites remains challenging due to the variation of GPP across species, structural features, and latitudinal locations.

Remote sensing (RS) provides the opportunity to characterize the ecosystem structures and environmental conditions and therefore, estimate the productivity of the ecosystems^3^. The light use efficiency (LUE) model was widely adopted to estimate GPP^4,5^. Currently, GPP models for terrestrial forest are applicable on a global scale (e.g., C-fix, MOD17, and GLO-PEM)^6–9^, however, production models have not been evaluated and employed in mangrove forests in a large scale, mainly due to the lack of understanding of carbon exchange in mangrove forests and measurements from flux tower.

Compared to terrestrial ecosystems, mangrove ecosystems are periodically inundated by the tides which contribute to the waterlogged and high salinity soil environment. Although mangroves have developed special structures or tissues to adapt to such demanding surroundings such as the aerial root, thick canopy, and salt-tolerance tissues, the environmental stresses remain critical to mangrove productivity. In addition to being affected by air temperature (T_air_) and vapor pressure deficit (VPD) as terrestrial forests^10,11^, mangrove forests are also influenced by the sea surface temperature (SST), salinity, and photosynthetic active radiation (PAR). Firstly, soil temperature affects the roots and aboveground metabolism of mangroves^12^. The high soil temperature would increase the respiration rate (R_e_). To minimize the water loss and energy consumption, the stomatal conductance within the mangrove would be reduced, which could lower the mangrove light saturation point (LSP) and constrain the photosynthesis. The low soil temperature may freeze the water-conducting xylem vessels of mangrove and therefore limit photosynthetic activities^13^. Therefore, apart from T_air_, the soil temperature may exert considerable impacts on mangrove photosynthesis. However, soil temperature in mangrove forests is difficult to measure due to tidal influence, but it has been reported that soil temperature is mostly related to SST due to tidal inundation^12,14^. Therefore, SST can be used to represent soil temperature and to investigate its effect on mangrove productivity. Secondly, the salinity of surface water and porewater represents a significant control on the mangrove LUE which is strongly related to the sea surface salinity, rainfall, and river discharge^14,15^. The high salinity leads to the negative osmotic pressure in the environment of roots which limits the water supply, and therefore inhibits the photosynthesis net photosynthetic rate^16,17^. Thirdly, the relatively low LSP makes the mangrove easy to reach light-saturated status^18^. Hence, the high PAR condition would bring excess light absorption and heat to the canopy that reduces the LUE of the canopy. These typical environmental stressors are not well understood and quantified. Studies have typically focused on the seasonal dynamics and interannual variation of carbon fluxes through modeling GPP^18–21^ or tidal effects on CO_2_ exchange based on in-situ measurements^22–25^. Barr et al.^14^ provided a satellite-driven model for estimating CO_2_ uptake in mangroves in the Florida Everglades, USA. For the first time, the effect of salinity on the mangrove LUE was investigated. Lele et al.^26^ proposed a vegetation photosynthesis model which can be well applied to relatively small-scale mangrove forest by incorporating in-situ LUE and high-resolution environmental scalars. However, the LUE models developed in their studies focused on the mangrove forests at a local scale, determining environmental parameters based on in-situ measurements. It is challenging to apply their models to other regions without continuous measurements.

Currently, there are no RS-based productivity products for mangrove forests globally. Therefore, scaling up carbon fluxes from flux tower to national and global scales considering the coastal environment is of great importance and challenge. Modeling the GPP of mangrove forests provides the first step in using RS to discover the role of mangrove ecosystems in global carbon budgets.

Therefore, the objectives of this study are (1) to improve the LUE model for mangroves considering environmental stressors in coastal zone (SST, salinity, and PAR), (2) to estimate the GPP of mangroves in the whole coastal zone of China combining flux tower-based measurements and RS, (3) to analyze the spatiotemporal distributions of mangrove productivity and the possible affecting factors.

## Results

### Effects of environmental stressors on mangrove LUE

#### Maximum LUE (LUE_max_)

**Figure 1:**
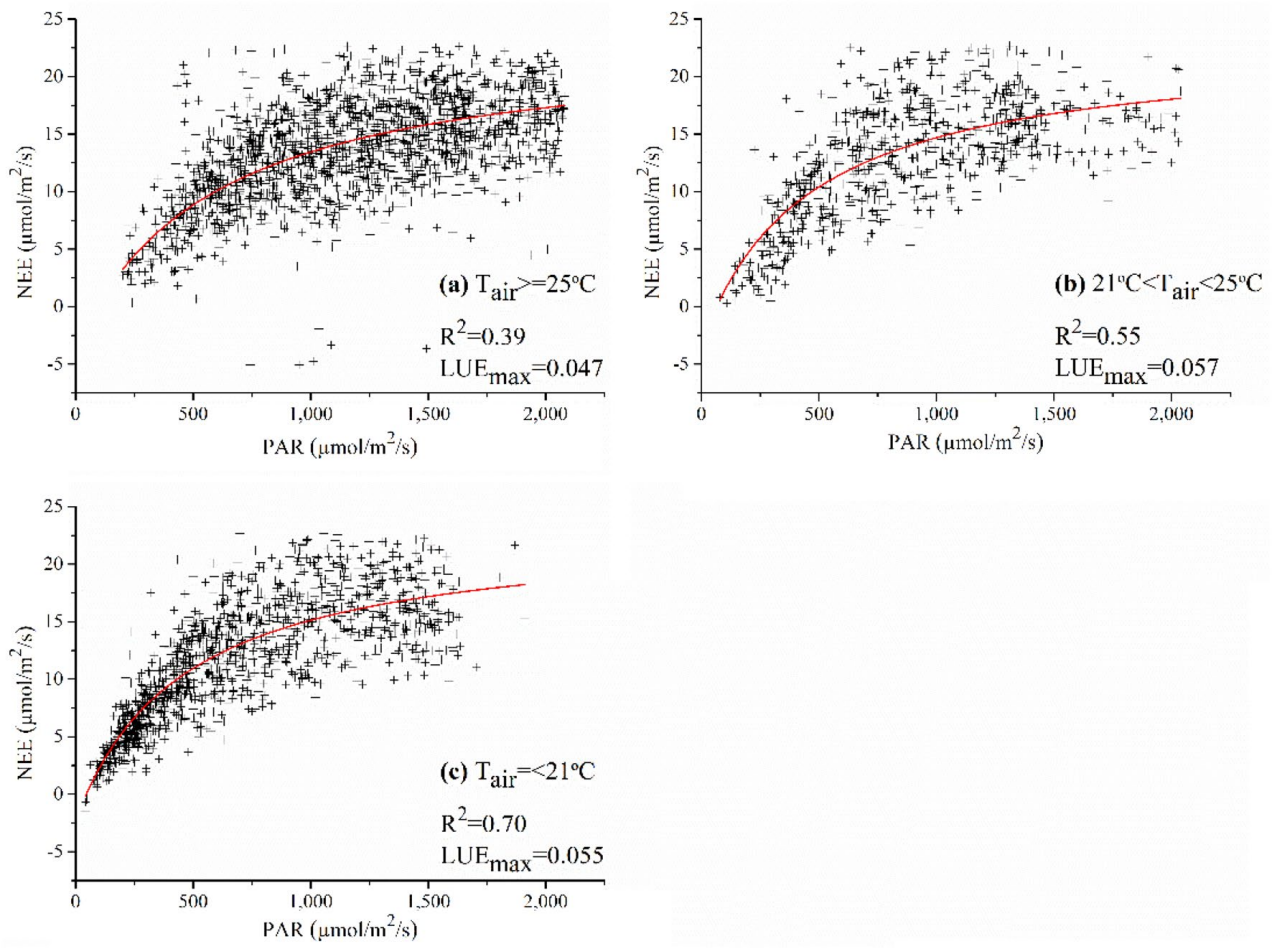
The responses of NEE to PAR at: (**a**) T_air_ ≥ 25 °C, (**b**) 21 °C  < T_air_ < 25 °C, and (**c**) T_air_ <  = 21 °C.

The maximum GPP (GPP_max_) and R_e_ for two mangrove forests are summarized in Table 1 based on the flux tower data. The net ecosystem exchange (NEE)-PAR fitted curves are displayed in Fig. 1. The initial slope of each rectangular hyperbola was calculated as the LUE_max_ and listed in Table 1. When the T_air_ was the most suitable for mangroves growth (21–25 °C), the LUE_max_ has the highest value (0.057); when the T_air_ was high (> 25 °C), the LUE_max_ was the lowest. We adopted the LUE_max_ for mangroves within the optimum T_air_.

**Table 1:** Parameters of three nonlinear hyperbolic models.

	T_air_	GPP_max_	R_e_	LUE_max_	R^2^	Number of observations
	(°C)	(μmol/m^2^/s)	(μmol/m^2^/s)	(mol C/mol PPFD)		
(a)	≥ 25	27.13	3.76	0.047	0.39	1893
(b)	21–25	26.12	3.22	0.057	0.55	790
(c)	≤ 21	25.45	2.26	0.055	0.70	1385

#### T_air scalar_ and SST_scalar_

The diurnal relationships between GPP and T_air_ were similar among four seasons, which were displayed in Fig. S1. The GPP typically increased with the increasing T_air_ and attained its maximum at noon. The highest GPP occurred when the T_air_ was around 25 °C, while the lowest T_air_ and the highest T_air_ were around 10 °C and 32 °C. The empirical values of T_min_, T_max_, T_opt_ from previous studies were summarized in Table S1. In general, mangroves cannot adequately develop when the mean T_air_ is below 10 °C, which corresponds with the SST around 12 °C during the coldest time of the year. While mangroves are intolerant to freezing temperatures below 0 °C for both T_air_ and SST^17^. Photosynthetic activities of most mangroves are strongly restricted when the T_air_ exceeds 35 °C^27^ and SST is over 32 °C. The optimal T_air_ is remarkably similar to the previous estimate of about 28 °C. Based on the literature review and in-situ data analysis, we ultimately adopted 10 °C, 28 °C and 35 °C for T_min_, T_opt_, and T_max_ and 12 °C, 24 °C, and 32 °C for SST_min_, SST_opt_, and SST_max_, respectively.

#### VPD_scalar_

Table S2 lists the VPD values in global mangrove forests from previous studies. VPD_max_ in mangrove forests ranged from 1.15 to 4.5 kPa, and VPD_min_ was around 0.09 to 1.18 kPa. Most mangroves grew properly at VPD values of between 0.44 and 1.37 kPa^11,28,29^, so finally, we adopted the VPD values of 0.6 kPa and 4 kPa as VPD_min_ and VPD_max_ to parameterize the VPD_scalar_ for mangrove ecosystems.

#### Salinity_scalar_

Figure S2 compared the RS-based salinity with in-situ salinity and daily rainfall. From this figure, we can clearly see that RS-based salinity differed significantly from the measured salinity. Barr et al.^30^ found that the surface water salinities above 28 ppt result in reduced NEE and LUE of mangroves. As the surface water salinity is usually lower than the sea water salinity due to the rainfall and river discharge, we assumed that surface water salinity is lower than 28 ppt if the RS-based sea water salinity is below 28 ppt. Therefore, a constraint was added to the calculation of Salinity_scalar_, which is equal to 1 when the salinity is below 28 ppt.

#### PAR_scalar_

**Figure 2:**
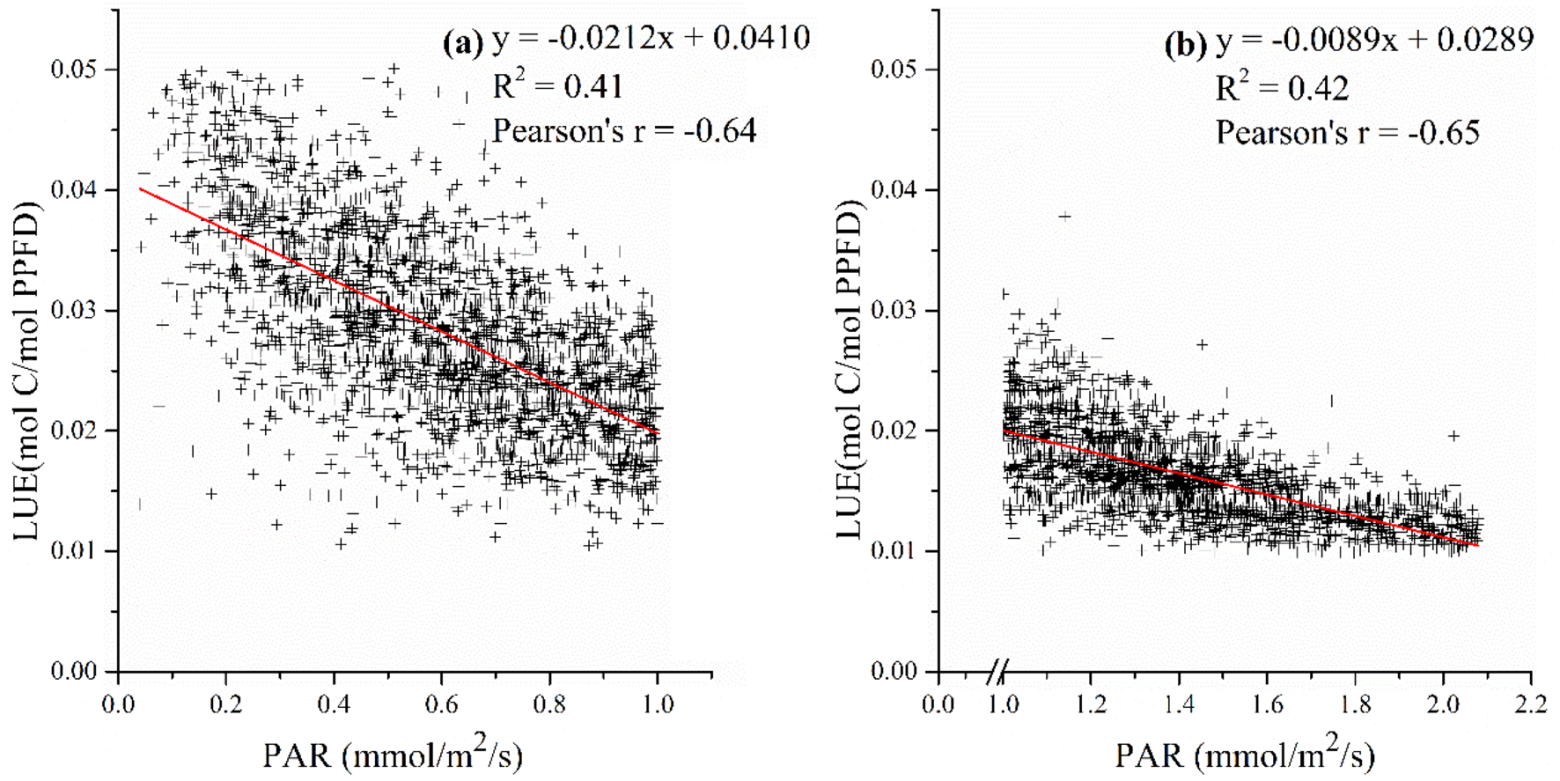
The response of LUE to PAR: (**a**) PAR =  < 1 mmol/m^2^/s and (**b**) PAR > 1 mmol/m^2^/s.

Barr et al.^14^ adopted a linear function to account for the photosynthesis saturation in mangrove LUE model. We took it as reference and investigated the light saturation effect in mangrove forests. Based on the responses of LUE to half-hourly PAR, we found that LUE declined with the increasing PAR as shown in Fig. 2. However, the decreasing rates changed with the increase of PAR. When PAR values were less than 1 mmol/m^2^/s, the decreasing rate was high. While when PAR exceeds 1 mmol/m^2^/s, the decreasing rate became lower. Therefore, we set the threshold of PAR to get the decreasing rate of PAR_scalar_. The m_par_ for PAR ≤ 1 mmol/m^2^/s and PAR > 1 mmol/m^2^/s were 0.5171 mmol/PAR and 0.3080 mmol/PAR.

### LUE validation

**Figure 3:**
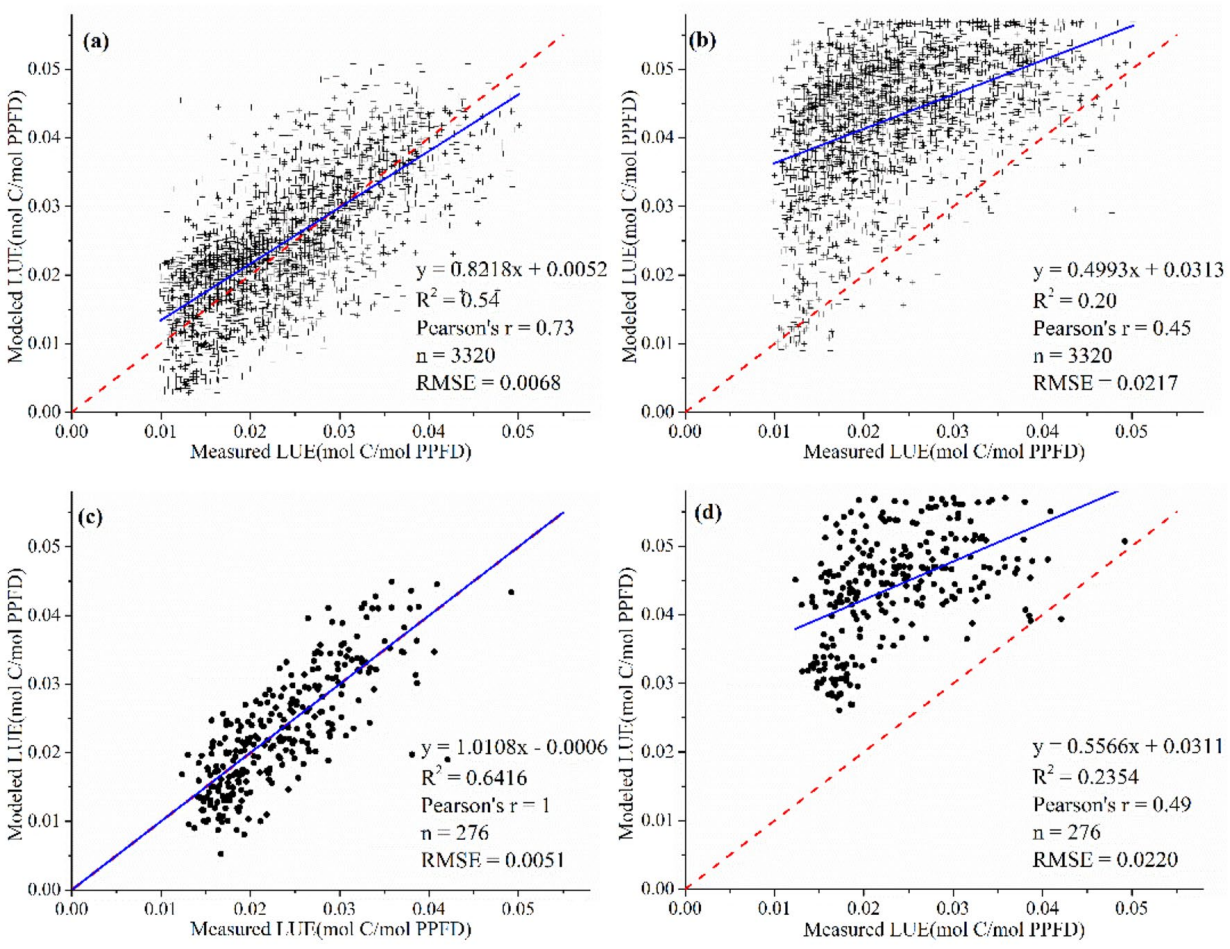
Validation of LUE estimated from terrestrial (**b**,**d**) and mangrove model (**a**,**c**) with daily- (**c**,**d**) and hourly-scale (**a**,**b**) data.

Figure 3 shows the validation results of the terrestrial MOD17 model and mangrove LUE model considering coastal environments, with hourly data (Fig. 3a,b) and daily data (Fig. 3c,d). LUE estimated by the mangrove model had higher accuracies with lower RMSE. LUE estimated with hourly meteorological data exhibited similar results and accuracies with the ones using daily scale data.

Figure S3 assessed the performance of each newly introduced variable on estimating LUE. Results showed that the LUE estimated with SST_scalar_ and Salinity_scalar_ maintained low accuracies with RMSE = 0.0185–0.0203, R^2^ = 0.2264–0.2332, and Pearson’s r = 0.48–0.49. However, PAR_scalar_ performed well in estimating LUE and exhibited a high consistency with the measured LUE (RMSE = 0.0048, R^2^ = 0.7147, and Pearson’s r = 0.85).

### GPP validation

**Figure 4:**
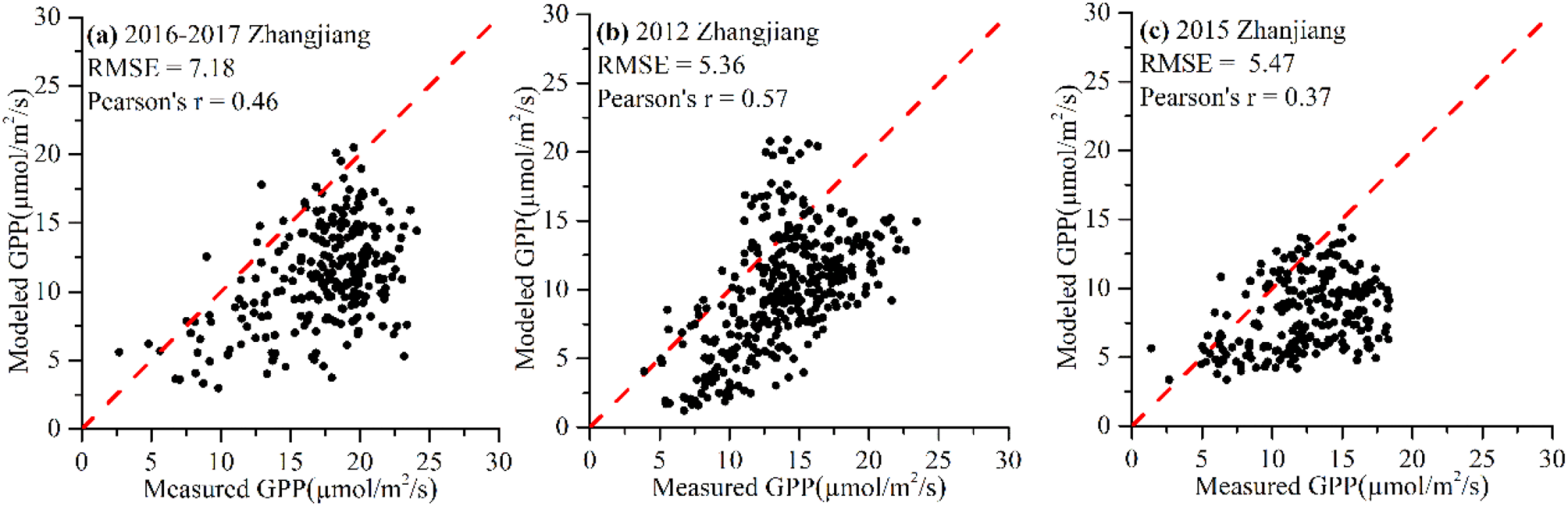
Validation of modeled GPP with flux-tower data from (**a**) Zhangjiang (2016–2017), (**b**) Zhangjiang (2012), and (**c**) Zhanjiang (2015).

**Figure 5:**
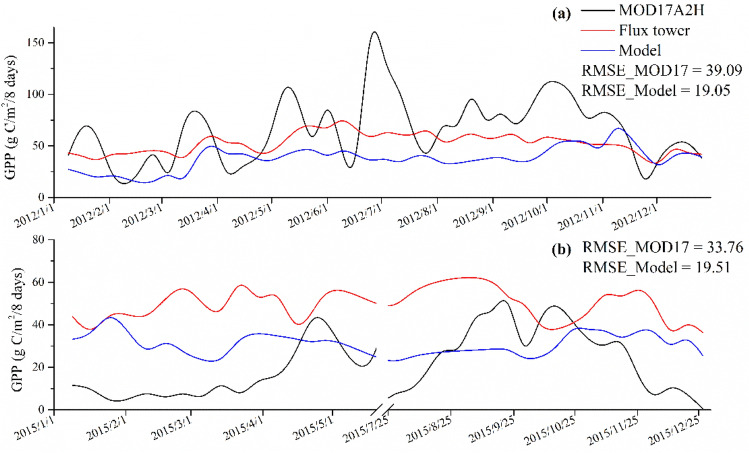
Time-series GPP comparisons among MODIS products, in-situ measurements, and mangrove GPP model generated in this study: (**a**) Zhangjiang (2012) and (**b**) Zhanjiang (2015).

The validation results for the mangrove GPP model are shown in Fig. 4. The results reveal that the GPP has relatively high accuracies with Pearson’s r around 0.5 and RMSE less than 7 μmol/m^2^/s. The GPP estimated was generally lower than the measured value. Figure 5 compares the time-series GPP results from MODIS, flux tower measurements, and model estimations. GPP estimated from our model had similar trends with the measured values, while MODIS GPP products have larger fluctuance. Modeled GPP had higher accuracies compared with MODIS GPP products which improved the RMSE from 39.09 to 19.05 g C/m^2^/8 days in 2012 and from 33.76 to 19.51 g C/m^2^/8 days in 2015.

### Spatiotemporal distributions of GPP

**Figure 6:**
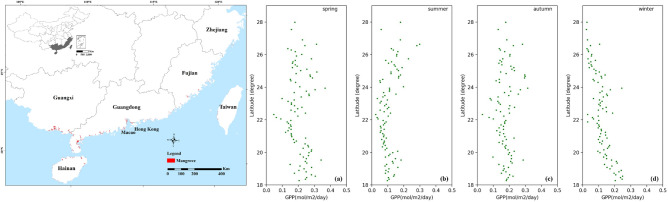
Seasonal and spatial variations of GPP in the whole mangrove forests in 2018: (**a**) spring, (**b**) summer, (**c**) autumn, and (**d**) winter (the coastal map was generated by ArcMap 10.8 with the license from Center for Spatial Information Science, The University of Tokyo; The China provincial administrative boundary data were downloaded from Resource and Environmental Science and Data Center; The mangrove distributions were from Zheng and Takeuchi^31^).

The seasonal and spatial distributions of GPP along the coastline are illustrated in Fig. 6. The overall GPP values were similar in all four seasons, fluctuating between 0.1 and 0.2 mol/m^2^/day, with slightly lower values in summer, mostly below 0.1 mol/m^2^/day, and higher values in spring and autumn, with average values around 0.2 mol/m^2^/day. In winter, GPP values were significantly lower in the high latitude zone, mostly below 0.1 mol/m^2^/day. At low latitudes, GPP increased with the decreasing latitude, especially below 20° N, up to 0.25 mol/m^2^/day.

**Figure 7:**
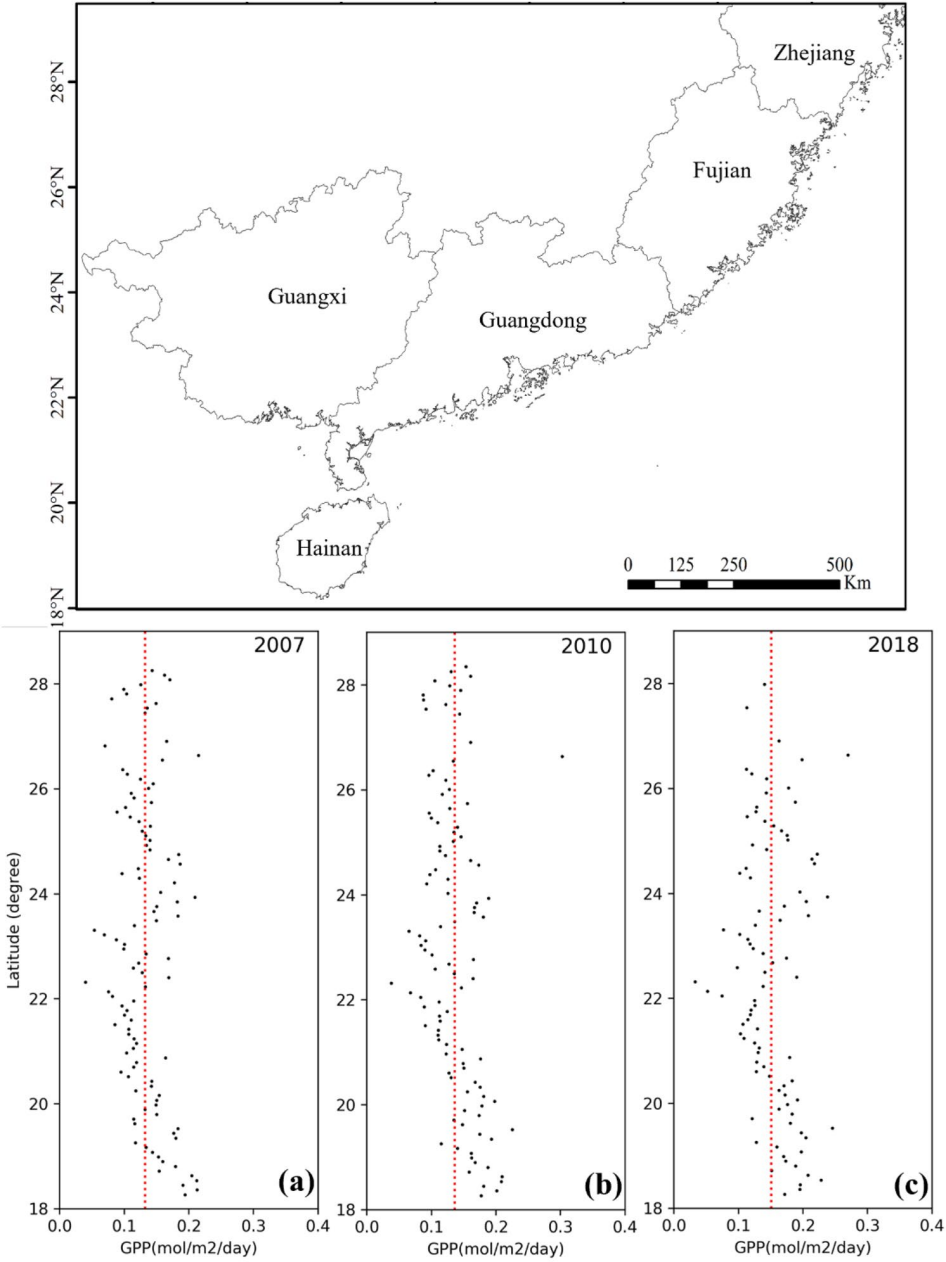
Spatiotemporal distributions of GPP in mangrove forests over China: (**a**) 2007, (**b**) 2010, and (**c**) 2018.

Figure 7 displays the spatiotemporal distribution of GPP for 2007, 2010, and 2018. Overall, GPP increased from 2007 to 2018, with average values of 0.13 mol/m^2^/day, 0.14 mol/m^2^/day, and 0.15 mol/m^2^/day for 2007, 2010, and 2018, respectively. Along the coastline, the spatial variations of GPP were similar. GPP tended to increase with the latitude below 22°N, reaching a maximum GPP of about 0.2 mol/m^2^/day at 18°N.

## Discussion

The improved performance of the mangrove LUE model considering coastal environments in this study was mainly attributed to the determination of environmental scalars. Parameters determining environmental stressors (e.g., T_opt_, T_min_, T_max_, VPD_min_, and VPD_max_) were set based on the general characteristics of mangroves worldwide. It may not be as accurate for the mangroves in our study sites, but it generally reflects the response of mangroves to environmental changes. Furthermore, as can be seen in Fig. S1, it is applicable to our study sites. Despite the specific characteristics of each mangrove ecosystem at different sites being preferred, this study first offers the possibility to estimate mangrove productivity at a larger scale to track GPP, thus emphasizing the role of mangrove ecosystems nationally or worldwide.

The validation results showed that the LUE values of the mangrove model agreed well with those estimated by EC method (Fig. 3) and indicated improved performance (slope = 0.8218–1.0108, intercept = -0.0006–0.0052, R^2^ = 0.54–0.64, RMSE = 0.0051–0.0068, Pearson’s r = 0.73–1), compared to the MOD17 model (slope = 0.4993–0.5566, intercept = 0.0311–0.0313, R^2^ = 0.24–0.45, RMSE = 0.0217–0.0220, Pearson’s r = 0.45–0.49). Firstly, the RS-based LUE model for terrestrial ecosystems (MOD17) considers only the environmental stressors of T_air_ and VPD. The photosynthesis in mangrove forests is influenced by other unique environmental factors caused by tidal inundation. According to Fig. S3, PAR caused the most significant effect on LUE, which is consistent with previous studies^14,30,32^. The impact of SST has not been quantitatively assessed, however, SST is a critical control that determines the upper latitudinal range of mangrove ecosystems^12,33^. In our study, the effects of SST and salinity on the mangrove LUE were quantified and helped improve LUE modeling.

Secondly, LUE_max_ was typically defined for different land covers, however, there were no specific values for mangrove forests. In this study, the LUE_max_ of mangroves was first determined. It is worth noting that daytime NEE responses to PAR vary depending on the T_air_^23,30,34^ so that LUE_max_ was determined separately at high, optimal, and low temperatures. The results showed that LUE_max_ reached a maximum when T_air_ was within the optimal range for mangroves, which represents the high productivity of mangrove ecosystems. Furthermore, the estimated LUE_max_ of mangrove forests (0.057) was larger than most terrestrial forests^35–37^, which could contribute to the high production and carbon sequestration in mangrove forests.

Lastly, the relatively low stomatal conductance of mangroves leads to low LSP compared with terrestrial forests, which could result in the high-irradiance stress for photosynthesis^38,39^. Mangrove LSP ranges from about 0.2–1.2 mmol/m^2^/s, depending on the species and environments^40–42^. LUE was relatively low in April and May when seasonal PAR was high, as photosynthesis is more likely to reach saturation. Therefore, we assumed the LUE of mangroves decreased with increasing PAR. In addition, we found that the downscaling effect of PAR on LUE was not constant, but varied with increasing PAR. As follows, different PAR scalars were set for mangroves according to different PAR values. This is a first attempt at refining PAR_scalar_ considering different solar radiation, which represents a significant departure from the assumption of a constant downscaling effect of PAR in RS-driven models^14,43^. The accuracy of the LUE model was improved by refining the PAR_scalar_ with different downscaling slopes, especially in periods of high PAR values.

Compared with the results obtained from flux-tower measurements, the modeled GPP was basically within the confidence interval of the measured results. The annual averages of GPP in Zhangjiang were 1729 g C/m^2^/year and 1924 g C/m^2^/year, in 2012 and 2016, and the annual mean value of GPP in Zhanjiang was 1434 g C/m^2^/year in 2015. The previous study showed that the GPP in Zhangjiang ranged from 1763 to 1919 g C/m^2^/year with a mean value of 1871 g C/m^2^/year^32,44,45^, which is in good agreement with the estimated values obtained in this study. Liu and Lai^46^ reported that the GPP of the Mai Po mangrove reserve was 2827 g C/m^2^/year. Rodda, et al.^20^ found a GPP value of 1271 g C/m^2^/year for Sunderbans mangroves in India. Gnanamoorthy, et al.^47^ estimated a GPP of 2305 g C/m^2^/year for Pichavaram mangroves. Variations in these estimates across sites were possibly caused by different climate-hydrological conditions, mangrove species, and ages. Differences in the same location may be due to different time scales and different methods of data gap filling and flux partitioning.

In a similar way to the GPP model for terrestrial ecosystems^48^, the effect of the mangrove GPP model on the accuracy of GPP estimates can vary considerably under different environmental conditions. However, in comparison with the accuracy of models built for other vegetation types, the GPP model in this study performed substantially in two sites with RMSE of 2.54–3.41 g C/m^2^/day. Wang et al.^49^ adopted different models to estimate GPP for global vegetation and validation results showed the RMSE ranged from 1.79 to 2.33 g C/m^2^/day. Xiao, et al.^50^ demonstrated that the deviation between observed and predicted GPP was about 35–282 g C/m^2^ in an evergreen needleleaf forest. Also, the absolute GPP errors were 7.94–20.92% and 9.97–13.70% for maize cropland and degraded grassland^36^. Despite the discrepancy, our results were generally consistent with previous studies and were verified by field observations near the flux towers.

The comparison of MODIS GPP and EC-estimated GPP showed that the MODIS GPP had a large fluctuation and weakly reflected productivity, being overestimated in 2012 and underestimated in 2015. Different meteorological inputs, different environmental scalars and fraction of absorbed photosynthetic active radiation (fAPAR) products in MODIS GPP and our mangrove GPP model can explain their different results. However, the improvements in our GPP model may help to obtain more accurate GPP estimates. The response of mangrove productivity to T_air_ has not been well-calibrated in the MODIS GPP product, which may partly account for the poor correlation between the MODIS GPP and EC estimates. Besides, MODIS GPP product was developed based on the International Geosphere-Biosphere Programme (IGBP) land cover map, which doesn’t include mangroves as a specific land cover^37^. Therefore, LUE_max_ and environmental parameters were not defined for mangroves, which varied with different environments. This may lead to uncertainty in MODIS GPP product for mangrove forests^14^. However, the GPP model generated in our study showed similar trends to the field measurements, capturing seasonal variations. The increase in the difference between MODIS GPP and EC estimates may be due to the assumption that the increase in GPP is linear with respect to PAR. In our model, the response of GPP to PAR was suppressed, resulting in seasonal changes in GPP that better match the observations. In addition, the GPP derived from this study was in higher agreement with measured values compared with GPP estimated from the vegetation photosynthesis model (VPM), as shown in Fig. S4. The improvement of this model was more obvious in winter (December to February), which may be due to the environmental stress of SST and PAR. The VPM without considering SST_scalar_ and PAR_scalar_ overestimated GPP in winter. It is indicated that the performance of the mangrove GPP model in this study varied with season. It is recommended to improve the estimation of GPP in the future by considering the seasonal variation of mangrove forests when determining environmental variables.

Most studies provide EC-based estimates of GPP that are measurements from a limited footprint. It is possible to extrapolate results across similar vegetation types and geographic settings, but not to areas of heterogeneous vegetation. The RS-based GPP model offers spatial-scale estimates that can be directly incorporated into ecosystem-type models. PAR, SST, and salinity are the key environmental parameters of this RS-based mangrove GPP model. SST and salinity data were derived from the satellite images, while PAR was generated from the reconstructed PAR data, since it is more accurate than the existing RS data and has historical year data. However, PAR products from Hamawari-8, MERIS, and SeaWiFS are available now, which provide an opportunity to obtain large-scale PAR data using RS in the future. In addition to this, GPP of two mangrove forests was assessed and validated with three-year measurements. Validation at different sites and years showed similar results, which indicated the model has similar performance across mangrove forests. Nonetheless, these estimates need to be corroborated with EC databases, which are relatively accurate and provide many additional variables that are currently beyond the scope of higher spatial-resolution RS estimates. The proposed GPP model considering coastal environments was well suited to extend the study area by incorporating RS information and meteorological data. Currently, there are still few mangrove carbon flux towers worldwide. The LUE and GPP models proposed in this study are difficult to validate with measurements from flux towers in other countries. However, local measurements are available in many countries with large mangrove forests, such as Thailand, Vietnam, India, and Bangladesh. Therefore, it is expected that comparisons with measurements from previous studies can be conducted to show the consistency and applicability.

The LUE model considering the effects of SST, salinity, and PAR performed well, however, the GPP estimated from the LUE, fAPAR, and PAR showed discrepancies and were generally lower than the measured values. Although the results are better than MODIS GPP products, limitations exist still.

Firstly, the effects of salinity and SST on mangrove productivity were directly related to tidal activities. The soil pore water and surface water salinity could affect the osmotic pressure of mangroves especially for the submerged parts which would control the stomatal conductance. In the same way, SST could influence the temperature of mangrove root systems and soil sediments which has impacts on mangrove roots’ respiration and transpiration. Although, theoretically, salinity and SST should be considered as environmental variables affecting mangrove LUE, our results (Fig. S3) indicated that salinity and SST have little influence on mangrove productivity^51^. To date, the quantitative impact of SST has not been comprehensively unfolded, but it is a global control that determines the upper limit of the latitudinal range of mangroves^12,33^. The weak relationships between salinity, SST, and mangrove GPP could be due to the uncertainty caused by tidal inundation. Tide duration, tide height, and tide cycle would determine the effect of salinity and SST on the mangrove LUE and GPP. However, quantifying the influence from the tidal cycle remains a challenging task, which could result in the relatively poor performance of Salinity_scalar_ and SST_scalar_ as shown in Fig. S3. Quantifying the soil temperature and surface water salinity considering the tidal cycle will contribute to model the LUE and GPP of mangrove forests.

Secondly, mangroves of different species and ages exhibit diverse structural and physical conditions, resulting in different LUE_max_, and optimal growing conditions such as T_opt_ and VPD_min_. The environmental settings would also vary from region to region. Liu and Lai^46^ found that LUE increased slightly with the increasing salinity below 15 ppt (R^2^ = 0.16). However, it was noted that photosynthetic activity of mangroves would be inhibited when the surface water salinity was high^30,51–53^. Probably, the mutual relationship between LUE and salinity depends on the salinity level and mangrove species. However, we have not specified the variables for different mangrove species, ages and locations which could be improved in the future. Besides, there are multicollinearities between different environmental variables. For example, T_air_ may have effects on SST and VPD, but as shown in Fig. S5, they are all important for mangrove photosynthesis. However, the correlations between them are not clear and need to be quantified in the future.

Thirdly, the relatively low spatial and temporal resolution of the environmental data from RS would influence the accuracy of the model. The datasets have a relatively coarse resolution (usually 500 m–1 km and daily) and are thereby less suitable for smaller nature reserves, especially in the narrow patches of mangrove areas that are rapidly being exploited in coastal China. Moreover, the variability in LUE decreases with increasing temporal scale^54^. In our study, we determined the PAR_scalar_ based on the response of LUE to hourly-scale PAR and found the different down-regulation effects with increasing PAR. However, this phenomenon is not obvious in previous studies. Most RS-based LUE models were developed at a daily or 8-day temporal scale^6,50,55–57^. In terrestrial forests, the light saturated effect caused by increasing PAR was neglectable with coarse temporal scale because the average PAR was usually lower than the LSP. However, as the time scale increases, the effect of light saturation on LUE becomes more pronounced^32,58,59^. More importantly, this effect is more obvious in mangroves due to their lower LSP^18,38^, which makes it important in mangrove LUE modeling. The results in Fig. 3 show similar performances of LUE model on hourly and daily scale. Thus, we suggested that our model can be adopted in hourly and daily temporal resolution. However, the PAR_scalar_ developed in this study was based on the mangrove forests in one study site which may be influenced by the mangrove species with different LSP and light conditions. What’s more, VPD was on a monthly scale, which cannot reflect environmental dynamics. However, the hourly and daily VPD data are currently not available for coastal areas in China. Therefore, we used monthly averages to represent daily VPD, which may lead to uncertainty in the derived GPP estimates (Figs. 6 and 7). Besides, porewater salinity is controlled by sea surface salinity, precipitation, and river discharge. However, currently, pore water salinity was expressed in terms of sea surface salinity, which may lead to an underestimation of Salinity_scalar_. More systematic study is necessary to make it more applicable and accurate on a large scale, of which modeling the LUE for different mangrove species and locations is inevitable. However, serving as a fundamental and preliminary step, our study aims to provide a framework for RS-based mangrove GPP modeling. Recently, with the advancement of satellite imagery, hourly-scale RS data for PAR, temperature and SST are available. It can be expected that our current work could be further improved by investigating the light saturation effects in different mangrove forests and adopt higher temporal resolution RS products such as Himawari-8 and GCOM-C in the future.

Lastly, the overall underestimation of GPP was mainly caused by the underestimation of fAPAR. Even though the fAPAR computed from Sentinel-2 had higher resolution and accuracy than MODIS fAPAR products, future improvements are still needed. Sentinel-2 fAPAR products (fAPAR-S2) was calculated as the instantaneous fAPAR obtained at 10:00 local solar time which only roughly represented the daily average but was not accurate. Besides, RS-derived fAPAR only considers the absorptions by living green vegetation elements, whereas the ground measured fAPAR refers to the contributions from all absorbing components^60^. The lower fAPAR-S2 values in mangrove forests may be due to the exposed-to-air root systems which absorb the radiation. Moreover, the spatial distribution of PAR was determined by Co-Kriging interpolation. The elevation was taken as the covariate to estimate spatial PAR. There are many other variables affecting the incoming PAR (e.g., slope and clearness)^61^. A more comprehensive set of variables needs to be included in the Co-kriging interpolation to improve the PAR estimation.

The spatial and seasonal variations of the mangrove GPP were related to environmental changes along the shoreline. The low summer GPP was explained by the lower fAPAR in summer compared with other seasons, which was principally due to the underestimation of fAPAR in summer. Furthermore, PAR_scalar_ took a mean value of LSP as 1 mmol/m^2^/s, however, LSP varied with different species and environmental conditions. In summer, mangroves are more likely to obtain light saturation, and thus PAR_scalar_ may lead to an underestimation of LUE and thus GPP. On the contrary, PAR values in winter were relatively low but increased slightly with decreasing latitude. Thus, the inhibitory effect of PAR on LUE was not significant, and GPP increased with decreasing latitude. Salinity and VPD were more stable across years and locations and had no noticeable effect on the mangrove LUE and GPP. The seasonal latitudinal patterns and effects on mangrove productivity were similar for T_air_ and SST. T_air_ and SST were lower in winter, especially at high latitudes where mangroves were more sensitive to cold weather. Therefore, the GPP of mangroves at high latitudes in winter was the lowest throughout the year. However, hot weather in summer also limited the photosynthesis in mangroves, especially at low latitudes, where T_air_ and SST were higher. Nevertheless, there were some correlations among these environmental constants. For example, the T_air_ affects the vapor pressure and SST. There was a positive correlation between PAR and T_air_. The multicollinearity among these variables and the various conditions of mangroves may affect the performance of the model and show variations along the coastline, which would be improved in future studies.

Additionally, the GPP of mangroves increased from 2007 to 2018, which was mainly due to the expansion of mangrove forests in the coastal areas. As mangroves grow, canopy size and tree density increase, which may lead to higher LUE and less underestimation of fAPAR, thus contributing to high productivity. However, Zhejiang province (27° 02′ N–31° 11′ N) experienced extremely cold weather in January 2016 caused by the East Asia cold wave^62,63^, and large areas of mangrove forests died or became sick, leading to a decline in the mangrove GPP at high latitudes in 2018.

## Conclusion

In conclusion, we presented a RS-based productivity model to estimate the GPP of mangrove forests in China. The model considered the environmental stresses induced by tidal inundation, therefore, involving SST, sea surface salinity, and PAR as environmental scalars to develop the LUE model. SST was first-ever included in the mangrove LUE model and parameterized by a similar model for T_air_. In addition, it was the first to indicate the different downscaling effects of PAR on the mangrove LUE with increasing PAR and determine the LUE_max_ under different T_air_. Consequently, the mangrove GPP was estimated based on the mangrove LUE model, fAPAR generated from Sentinel-2 images and reconstructed PAR from meteorological stations. The results revealed that PAR, T_air_, VPD, SST, and salinity are clearly drivers of diurnal and seasonal variations in the mangrove LUE and CO_2_ fluxes. Among them, PAR, SST, and salinity are unique to mangrove ecosystems. The LUE model developed for mangrove forests had higher overall accuracy (RMSE = 0.0051, R^2^ = 0.64) than the LUE model (MOD17) for terrestrial forests (RMSE = 0.0220, R^2^ = 0.24). GPP estimated in this study generally agreed with in-situ measurements from two carbon flux towers. Although there are still limitations, the modeled GPP maintained higher accuracies compared with MODIS GPP products. These results demonstrated the potential of RS-driven productivity models for the large-scale mangrove GPP estimation and provided fundamental data and scientific methodological support for future mangrove blue carbon potential assessment and restoration policy development.

## Materials and methods

### Study area

Two carbon flux towers have been established in Zhangjiang Estuary Mangrove National Nature Reserve (117° 24′ 53.02″ E, 23° 55′ 26.63″ N) in Fujian and Zhanjiang Mangrove National Nature Reserve (110° 09′ 44.67″ E, 20° 56′ 24.08″ N) in Guangdong (Fig. S6), China. The mangroves in Zhangjiang and Zhanjiang are mainly composed of *Kandelia obovate* and *Sonneratia apetala*. The forest structures and microclimate in these two sites were various and listed in Table S3.

### Site-specific data from carbon flux towers

Half-hourly carbon fluxes between the canopy and atmosphere were obtained from flux towers and processed by the EC method. Meteorological and tidal information was measured with multiple instruments near the flux tower. All data were provided by the ChinaFlux network (http://www.chinaflux.org). Table S4 summarizes the data availability. More details of the EC system structure and data processing can be referred to published papers^18,32^.

### Remote sensing data

Historical meteorological data were derived from different satellites or based on the reanalysis data. The summary of the dataset can be found in Table 2. Meteorological data were obtained from the Google Earth Engine platform. Sentinel-2 L1C images were adopted for computing the fAPAR. PAR was derived from the reconstructed PAR dataset^64^ which was derived from the meteorological data, MODIS AOD data and NASA/GSFC O_3_ data. Zheng and Takeuchi^31^ mapped the mangrove distributions in China for 2007, 2010, and 2018 which were used to determine the mangrove area.

**Table 2:** Summary of the climatic data.

	Dataset	Spatial resolution	Time resolution
T_air_	ERA5 reanalysis data	0.25°	Daily
VPD	TerraClimate	2.5 arc minutes	Monthly
SST	MODIS Aqua data	500 m	Daily
Salinity	Hybrid coordinate ocean modal (HYCOM)	0.08°	Daily
PAR	Reconstructed PAR	Point data	Daily

### Mangrove productivity estimation

#### LUE modeling

LUE was estimated based on the vegetation type and environmental stress as the function of LUE_max_ and environmental scalars, which is shown in Eq. (1)^43,55,65,66^.

1 $$LUE={LUE}_{max}\times {T}_{air \,\,scalar}\times {VPD}_{scalar}$$

where LUE_max_ is the maximum LUE, T_air scalar_ and VPD_scalar_ are the down-regulation scalars for the effects of T_air_ and water on LUE. However, the environmental factors affecting the mangrove photosynthetic metabolism have significant differences with terrestrial forests^13,30,67^. Although it is hard to isolate individual effects from these environmental factors without concurrent photochemical measurements, the diurnal and seasonal changing patterns revealed that the temperature (T_air_ and SST), PAR, VPD, and salinity represent the controlling factors of LUE variations on both diurnal and seasonal scales. Therefore, in this study, T_air_, SST, PAR, VPD, and salinity were considered as the environmental stressors for the mangrove LUE and corresponding scalars were defined to establish the LUE model, which was proposed as:

2 $$LUE={LUE}_{max}\times {T}_{air\,\, scalar}\times {VPD}_{scalar}\times {SST}_{scalar}\times {Salinity}_{scalar}\times {PAR}_{scalar}$$

where SST_scalar_, Salinity_scalar_, and PAR_scalar_ are the down-regulation scalars for the effects of SST, surface water salinity, and PAR on the mangrove LUE, respectively. The parameterizations of each environmental scalar are explained as follow:

#### LUE_max_

LUE_max_ describes the maximum efficiency of vegetation for fixing solar energy and is typically related to the chlorophyll content, leaf age, species, light intensity, and growth stages^68^. The LUE_max_ of MODIS GPP/NPP Project were derived for 11 biomes including evergreen needleleaf forest, evergreen broadleaf forest, deciduous needleleaf forest, deciduous broadleaf forest, mixed forest, closed shrubland, open shrubland, woody savanna, savanna, grassland, and cropland^37^. However, no existing LUE_max_ is available for mangrove forest. We adopted the nonlinear hyperbolic model (Michaelis–Menten function) as Eq. (3) to simulate the relationship between NEE and PAR which was widely used for terrestrial vegetations^36,55,69^.

3 $$NEE=\frac{{LUE}_{max}\times PAR\times {GPP}_{max}}{{LUE}_{max}\times PAR+{GPP}_{max}}-{R}_{e}$$

where GPP_max_ is the maximum GPP over a year, and R_e_ is the ecosystem respiration at night. LUE_max_ was determined by fitting the light response curves (NEE versus PAR) with the daytime half-hourly NEE and PAR values from the growing seasons of mangroves (September to February in Zhangjiang). As the responses of NEE to PAR were related to T_air_^30^, LUE_max_ may vary under different T_air_. LUE_max_ could reach the maximum when T_air_ was within the optimal range for mangroves, which represents high productivity of mangrove ecosystems. LUE_max_ should be relatively low at high and low temperatures. This is mainly because at high and low temperatures, mangroves are likely to reduce their stomatal conductance to lessen the transpiration, which may lower their LSP and make them more sensitive to solar irradiance. The optimal T_air_ for mangroves is 21–25 °C, so we set the T_air_ ranges as: T_air_ ≤ 21 °C, 21 °C  < T_air_ < 25 °C, and T_air_ ≥ 25 °C. LUE_max_ was determined for each temperature range and the maximum was adopted for LUE model.

#### T_air scalar_ and SST_scalar_

Mangrove photosynthesis is restricted to a certain optimum temperature range for T_air_ and SST. The low temperature may freeze the water-conducting xylem vessels of the mangrove, and the high temperature would reduce the stomatal conductance^13^. So, we assumed that the mangrove LUE increases with the increase of temperature, however, it will start to decrease after a certain value. Therefore, T_scalar_ was defined as Eq. (4) which was proposed by Raich, et al.^70^ for different vegetations.

4 $${T}_{air\,\, scalar}=\frac{(T- {T}_{min})(T- {T}_{max})}{\left(T- {T}_{min}\right)\left(T- {T}_{max}\right)-{\left(T- {T}_{opt}\right)}^{2}}$$

where T_min_, T_max_, and T_opt_ are minimum, maximum, and optimal air temperatures for the mangrove photosynthetic activities, respectively. If T is below T_min_, T_scalar_ is set to be zero. The daily mean temperature (T_mean_) and daily maximum temperature (T_max_) were employed to calculate the daytime air temperature following Eq. (5)^71^.

5 $$T=\frac{({T}_{mean}+{T}_{max})}{2}$$

Although T_air scalar_ was originally developed for the terrestrial forest, Barr et al.^14^ quantified it in the mangrove LUE model. In this study, we adopted the empirical values to estimate the T_max_, T_min_, and T_opt_ and then validated them with the GPP-T_air_ relationship based on the in-situ measurements. The photosynthetic responses of mangrove to SST are similar to T_air_, so we assume it follows the same function but unique characteristics (SST_max_, SST_min_, and SST_opt_). Therefore, the scalar for SST (SST_scalar_) was derived in the same way as T_air scalar_.

#### VPD_scalar_

VPD_scalar_ is saturated at both maximum and minimum VPD and can be calculated by Eq. (6)^37^.

6 $${VPD}_{scalar}=\frac{{VPD}_{max}-VPD}{{VPD}_{max}-{VPD}_{min}}$$

where VPD_max_ and VPD_min_ are the maximum and minimum daytime VPD. If VPD is less than VPD_min_, VPD_scalar_ is set to be 1. If VPD is larger than VPD_max_, VPD_scalar_ will be set as 0. We determined VPD_min_ and VPD_max_ by summarizing from previous studies and verified with in-situ data.

#### Salinity_scalar_

The decline in LUE with increasing salinity was quantified by Eq. (7)^14^.

7 $${Salinity}_{scalar}=1-Salinity\times {m}_{sal}$$

where m_sal_ represents the decreasing rate of Salinity_scalar_ in response to the increasing salinity. The m_sal_ was estimated at 0.0047 ± 0.0022^14^. Consequently, we employed m_sal_ as 0.0047 to determine the salinity scalar for mangrove ecosystems.

#### PAR_scalar_

A linear function in Eq. (8)^14^ was included to reflect the PAR constraint on mangrove LUE. It accounts for photosynthesis saturation manifested as declining LUE with increasing PAR.

8 $${PAR}_{scalar}=1-PAR\times {m}_{par}$$

where m_par_ represents the decreasing rate of PAR_scalar_ to the increasing PAR. The m_par_ was determined by the response of LUE to increasing PAR.

#### GPP modeling

**Figure 8:**
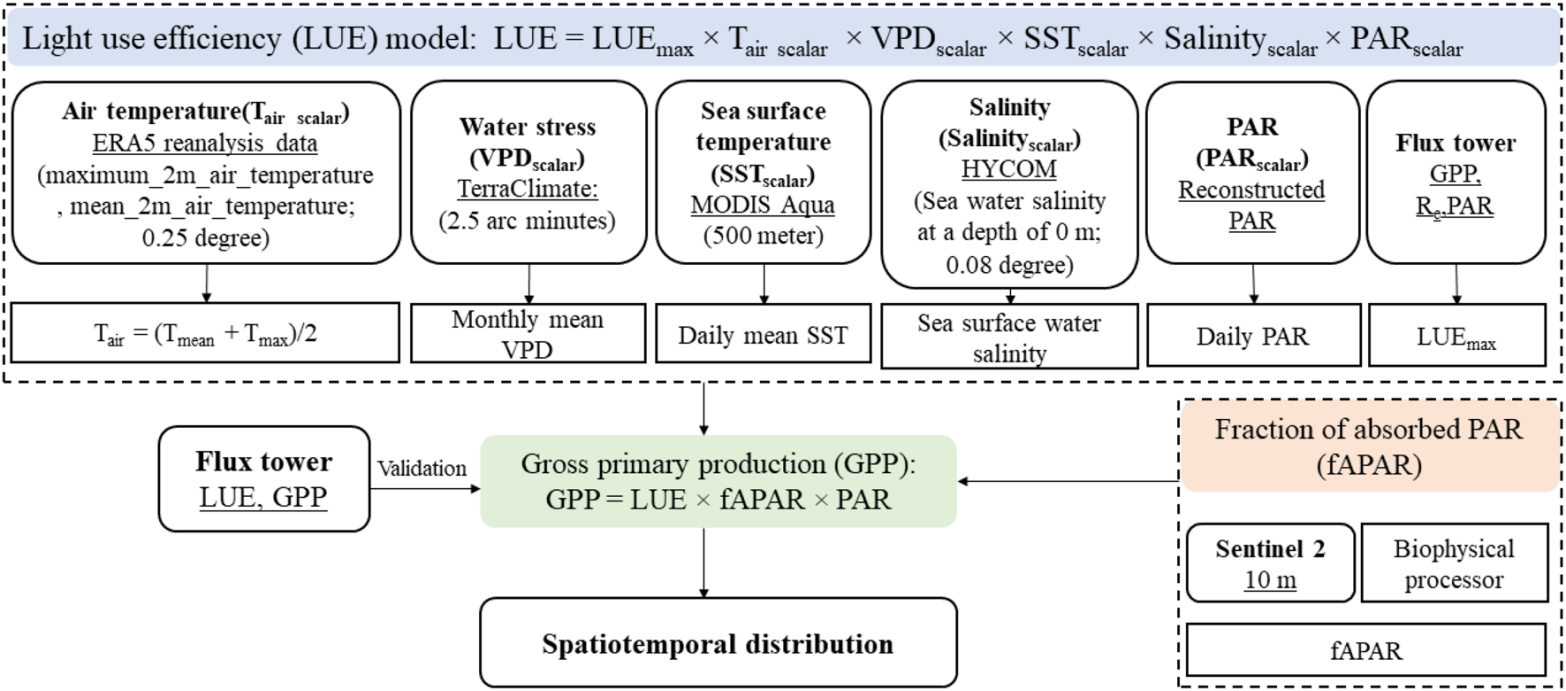
Overall flowchart of GPP modeling.

GPP was calculated as Eq. (9)^4,72^ and the overall flowchart can be summarized as Fig. 8:

9 $$GPP=PAR\times fAPAR\times LUE$$

Firstly, the LUE of mangroves in these two mangrove reserves was calculated based on the LUE model proposed before. Resampling of RS data was carried out to keep the spatial resolution at 500 m and the temporal resolution at daily. Since SST and salinity were derived from the sea surface, the nearest sea surface pixel to the mangrove was adopted to represent the effects of SST and salinity on mangrove LUE.

Then, fAPAR was computed using the biophysical processor in SNAP software^73^ by ANN^74–76^ trained with the RTM PROSAIL^77^. The overflow and key parameters for fAPAR estimation were summarized in Fig. S7. The processed fAPAR-S2 represents the daily integrated fAPAR values, following the assumption that the instantaneous fAPAR value at 10:00 (or 14:00) solar time is close to the daily integrated value under clear sky conditions^78^. Besides, outlier pixels in fAPAR-S2 were eliminated and only pixels with “QA = 0 0 0” were adopted.

After that, reconstructed PAR data were obtained from 724 meteorological stations provided by Tang, et al.^64^. The PAR data used were validated against PAR data measured in two mangrove reserves, showing good agreement, as displayed in Fig. S8. We further interpolated the PAR data from meteorological stations to the whole coastal zone by the Co-Kriging interpolation method^79^ taking surface elevation as covariate^80,81^. Finally, GPP in these two mangrove reserves was estimated based on the derived LUE, fAPAR, and PAR.

### Model validation and application

The LUE modeled with hourly and daily environmental data were validated with LUE values from the carbon fluxes tower in Zhangjiang mangrove reserve. In addition, LUE was estimated according to the MOD17 model for terrestrial forests considering only the effects of T_air_ and VPD, as shown in Eq. (1). Hourly and daily meteorological data were taken as inputs to validate the model on different time scales. The experimental results were compared with in-situ LUE to evaluate the performance. GPP estimated using the proposed model was validated with the flux tower measurements. The GPP derived considering coastal environments was in turn converted to a cumulative 8-day composite and compared with MODIS GPP product at the same resolution^82^.

After validation, the GPP model was applied to estimate the GPP of mangrove forests in the whole coastal zone of China for the years 2007, 2010, and 2018. Besides, seasonal variations were displayed to reflect the different productivity of mangroves under various environmental conditions.

## Supplementary Information

Supplementary Information.

## Data Availability

The datasets generated during and/or analyzed during the current study are available from the corresponding author on reasonable request.
