# Supplementary material for: Estimating mangrove forest gross primary production by quantifying environmental stressors in the coastal area
Source: Sci Rep. 2022 Feb 9;12:2238. doi: 10.1038/s41598-022-06231-6 (PMC8828879; doi:10.1038/s41598-022-06231-6)
Supplement: Supplementary file 1 — Supplementary Information. [file 41598_2022_6231_MOESM1_ESM.docx]

**Supplementary Information**


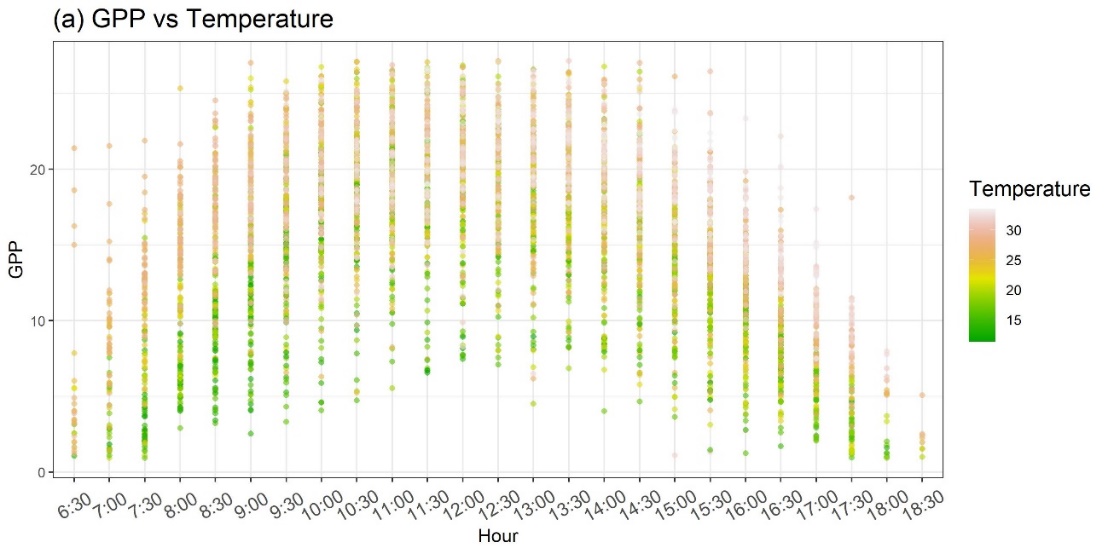


**Figure S1** The relationship between half-hourly T_air_ and GPP.





**Figure S2** Time series in-situ surface water salinity, satellite-based seawater salinity, and rainfall.





**Figure S3** Validation of LUE by individual environmental variables specified: (a) SST, (b) Salinity, and (c) PAR.





**Figure S4** Comparisons of GPP among flux tower measurement, mangrove model from this study, and vegetation photosynthesis model (VPM) in: (a) Zhangjiang 2012, and (b) Zhanjiang, 2015.


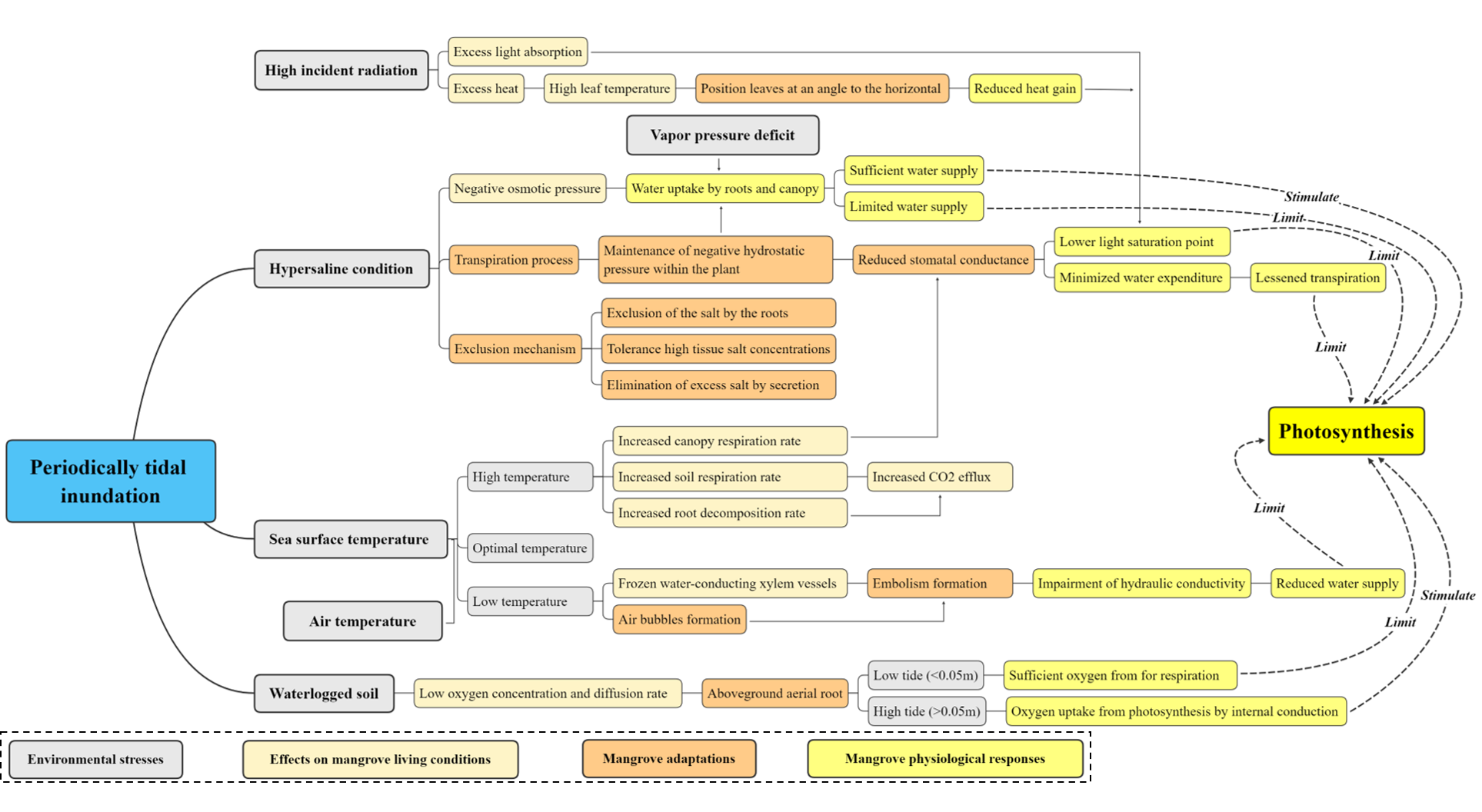


**Figure S5** Physical mechanisms of mangrove photosynthetic response to tidal inundation.


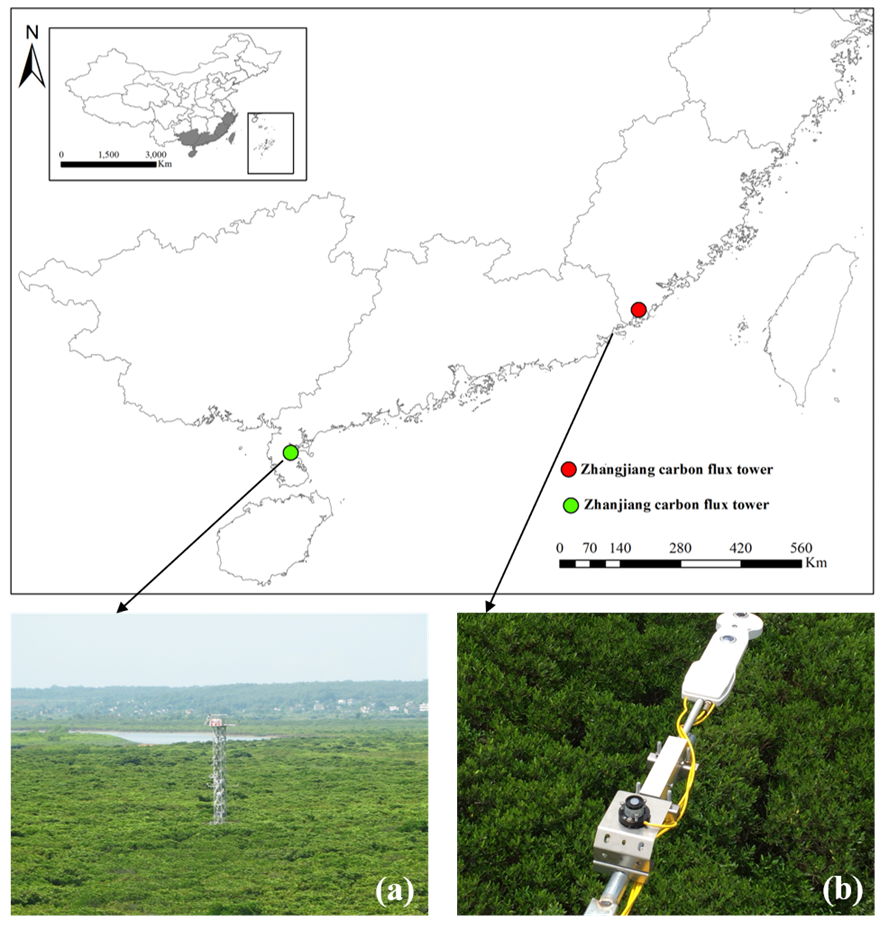


**Figure S6** Locations of carbon flux towers in China at: (a) Zhangjiang, and (b) Zhanjiang (Field photos from ChinaFLUX website: [http://www.chinaflux.org](http://www.chinaflux.org/index.aspx) ).


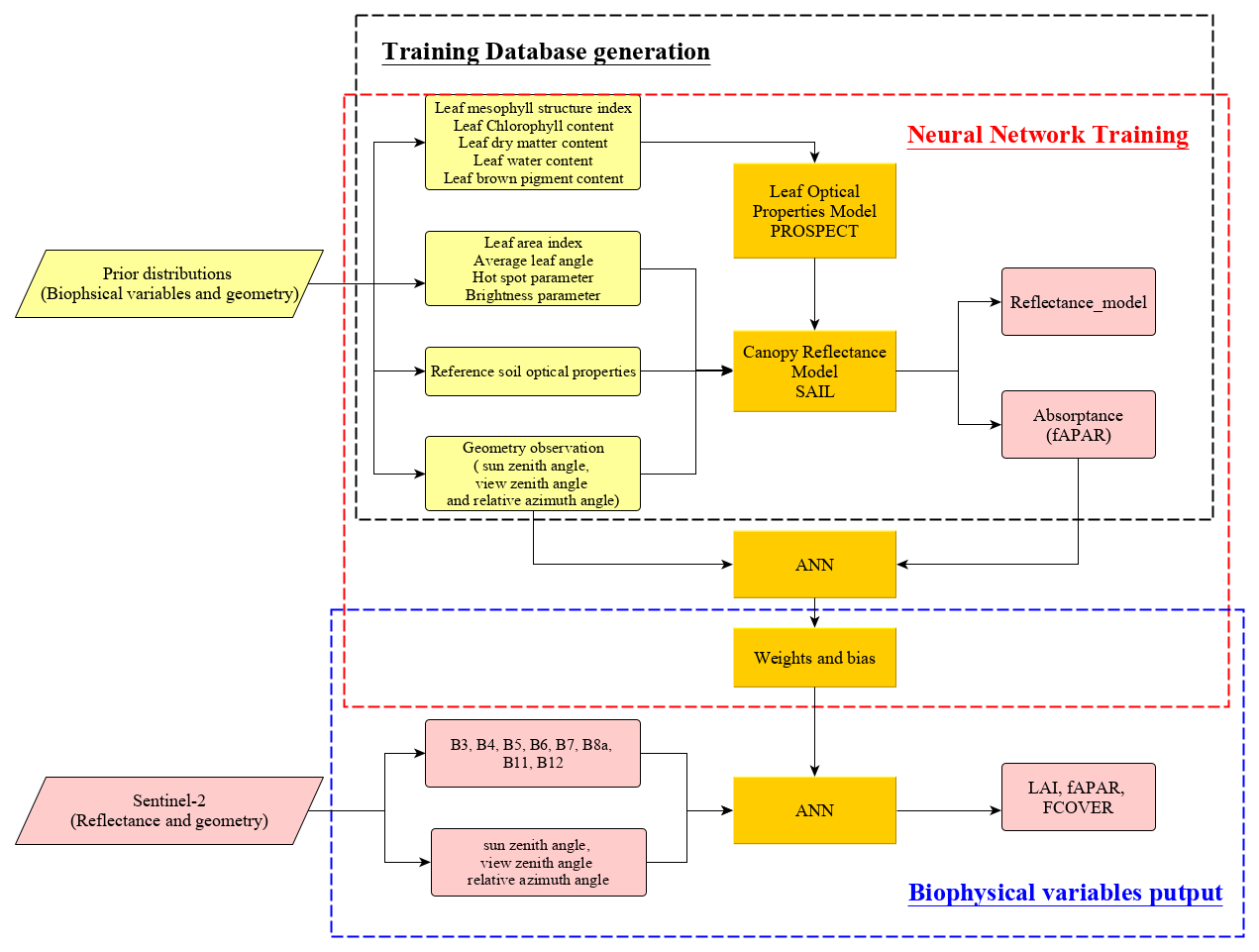


**Figure S7** Overflow for Sentinel-2 fAPAR modelling.





**Figure S8** Scatterplots between the daily flux-tower PAR and reconstructed PAR in: (a) Zhangjiang (2012), (b) Zhangjiang (2016-2017), and (c) Zhanjiang (2015).

**Table S1** Summary of T_min_, T_max_ and T_opt_ for T_air_ and SST based on literature reviews.

|  | **T_min_ (℃)** | **T_max_ (℃)** | **T_opt_ (℃)** | **Reference** |
| --- | --- | --- | --- | --- |
| Florida, United States (25.36° N, 81.08° W) | 2.6 | 33.5 | 27.8 | Barr, et al. ^1^ |
| New Caledonia (20.94° N, 164.66° E) | 10.5 | 34.4 | 26.8 | Leopold, et al. ^2^ |
| Trat River, Thailand (12.20° N, 102.57° E) | 24.0 | 32.1 | 27.6 | Poungparn, et al. ^3^ |
| Ningde, China (26.68° N) | 1.4 | -- | 19.0 | Chen, et al. ^4^ |
| Longhai, China (24.43° N) | 4.6 | -- | 21.0 | Chen, et al. ^4^ |
| Zhangjiang, China (23.90° N) | 6.5 | -- | 21.2 | Chen, et al. ^4^ |
| Shenzhen, China (22.53° N) | 5.3 | -- | 22.5 | Chen, et al. ^4^ |
| Gaoqiao, China (21.56° N) | 5.2 | -- | 23.0 | Chen, et al. ^4^ |
| Zhanjiang, China (21.00° N) | 3.8 | -- | 23.0 | Chen, et al. ^4^ |
| Fangchenggang, China (21.46° N) | 4.1 | -- | 22.5 | Chen, et al. ^4^ |
| Qinzhou, China (21.72° N) | 3.3 | -- | 22.0 | Chen, et al. ^4^ |
| Haikou, China (19.90° N) | 7.7 | -- | 23.8 | Chen, et al. ^4^ |
| Wenchang, China (19.57° N) | 9.5 | -- | 24.0 | Chen, et al. ^4^ |
| Global | -- | 33 | 30 | Alongi ^5^ |
| Yueqing, China (28.42° N) | 9.3 (T_air_)  10.6 (SST) | -- | -- | Huang, et al. ^6^ |
| Fuding, China (27.33° N) | 9.8 (T_air_)  10.9 (SST) | -- | -- | Zhang, et al. ^7^ |
| Global (*Avicennia* species) | 8.1 (T_air_)  12.7 (SST) | 35.6 (T_air_)  32.8 (SST) | -- | Quisthoudt, et al. ^8^ |
| Global (*Rhizophora* species) | 13.1 (T_air_)  16.4 (SST) | 34.6 (T_air_)  32.6 (SST) | -- | Quisthoudt, et al. ^8^ |
| Global | 19 | 35 | -- | Noor, et al. ^9^ |
| Global | 16 | 38-40 | 28-32 | Gilman, et al. ^10^ |

**Table S2** Summery of VPD_min_ and VPD_max_ in mangrove forests.

| Locations | VPD_min_ | VPD_max_ | Reference |
| --- | --- | --- | --- |
| Everglades National Park, USA (25.36° N, 81.08° W) | 0.16 | 2.23 | Barr, et al. ^11^ |
| North Peninsula State Park, USA (29.42° N, 81.10° E) | 0.15 | 2.20 | Devaney, et al. ^12^ |
| Old Point Mangrove Regional Park, San Andrés Island (12.58° N, 81.74° W) | 1.18 | 1.72 | Sánchez-Núñez and Mancera-Pineda ^13^ |
| North Stradbroke Island, Australia (27.45° S, 135.43° E) | 0.10* | 1.25* | Vandegehuchte, et al. ^14^ |
| Amelia Island, USA (30.67° N, 81.43° W) | 0.90* | 3.70* | Aspinwall, et al. ^15^ |
| Terranora Broadwater, Australia (28.22° S, 153.51° E) | 0.10* | 1.20* | Vilas, et al. ^16^ |
| Okinawa Island, Japan (26.18° N, 127.67° E) | 0.64 | 1.15 | Sharma, et al. ^17^ |
| Pichavaram, India (11.33° N, 79.92° E) | 0.10* | 4.50* | Gnanamoorthy, et al. ^18^ |
| Sinnamary, France (52.93° E, 5.39° N) | 0.10* | 1.25* | Muller, et al. ^19^ |
| Sundarban, India (20.53 - 20.67° N, 88.08 - 89° E) | 0.09 | 2.17 | Ganguly, et al. ^20^ |

*^Values extracted from figures by visual interpretation.^

**Table S3** Information on the mangrove forest structures and local climate.

| **Site** | **Mangrove forest** | | |  | **Microclimate (Annual mean)** | | | |
| --- | --- | --- | --- | --- | --- | --- | --- | --- |
|  | Height (m) | Species | Age  (yr) |  | T_air_  (°C) | humidity | Tide range (m) | Rainfall  (mm) |
| Zhangjiang | 6 | *Kandelia obovate*, Avicennia *marina*, *Aegiceras corniculatum* | -- |  | 21.2 | 79% | 2.3 | 1715 |
| Zhanjiang | 12 | *Sonneratia apetala* | >20 |  | 24.5 | -- | 3.0 | 1619 |

**Table S4** In-situ data availability.

| **Site** | **Carbon flux tower data** | | | | |  | **Meteorological data** | | | | |
| --- | --- | --- | --- | --- | --- | --- | --- | --- | --- | --- | --- |
|  | GPP | NEE | R_e_ | fAPAR | LUE |  | PAR | T_air_ | VPD | SST | Salinity |
|  | μmol/m^2^/s | | |  |  |  | mol/m^2^/s | °C | kPa | °C | ppt |
| Zhangjiang | Half-hourly, 2012.01-12, 2016.08-2017.08 | | Half-hourly, 2012.01-12 | Half-hourly, 2016.08-2017.08 | |  | Half-hourly,  2012.01-12,  2016.08-2017.08 | | | Half-hourly, 2016.08-2017.08 | |
| Zhanjiang | Half-hourly,  2015.01-2015.12 | | |  | |  | Half-hourly,  2015.01-2015.12 | | |  | |

* NEE = net ecosystem exchange; fAPAR = fraction of absorbed photosynthetic active radiation.

**References**

1 Barr, J. G., Engel, V., Fuentes, J., Fuller, D. & Kwon, H. Modeling light use efficiency in a subtropical mangrove forest equipped with CO 2 eddy covariance. *Biogeosciences* **10**, 2145-2158 (2013).

2 Leopold, A. *et al.* Net ecosystem CO2 exchange in the “Coeur de Voh” mangrove, New Caledonia: Effects of water stress on mangrove productivity in a semi-arid climate. *Agricultural and forest meteorology* **223**, 217-232 (2016).

3 Poungparn, S. *et al.* Ten-Year Estimation of Net Primary Productivity in a Mangrove Forest under a Tropical Monsoon Climate in Eastern Thailand: Significance of the Temperature Environment in the Dry Season. *Forests* **11**, 987 (2020).

4 Chen, L. *et al.* Mangrove species' responses to winter air temperature extremes in China. *Ecosphere* **8**, e01865 (2017).

5 Alongi, D. M. Impact of global change on nutrient dynamics in mangrove forests. *Forests* **9**, 596 (2018).

6 Huang, X., Peng, X., Qiu, J. & Chen, S. Mangrove status and development prospects in southern Zhejiang Province. *Journal of Zhejiang Forestry College* **26**, 427-433 (2009).

7 Zhang, Q. *et al.* Marine environmental indexes related to mangrove growth. *Acta Ecologica Sinica* **21**, 1427-1437 (2001).

8 Quisthoudt, K. *et al.* Temperature variation among mangrove latitudinal range limits worldwide. *Trees* **26**, 1919-1931 (2012).

9 Noor, T., Batool, N., Mazhar, R. & Ilyas, N. Effects of siltation, temperature and salinity on mangrove plants. *European Academic Research* **2**, 14172-14179 (2015).

10 Gilman, E. L., Ellison, J., Duke, N. C. & Field, C. Threats to mangroves from climate change and adaptation options: a review. *Aquatic botany* **89**, 237-250 (2008).

11 Barr, J. G., DeLonge, M. S. & Fuentes, J. D. Seasonal evapotranspiration patterns in mangrove forests. *Journal of Geophysical Research: Atmospheres* **119**, 3886-3899 (2014).

12 Devaney, J. L., Pullen, J., Feller, I. C. & Parker, J. D. Low humidity and hypersalinity reduce cold tolerance in mangroves. *Estuarine, Coastal and Shelf Science* **248**, 107015 (2021).

13 Sánchez-Núñez, D. A. & Mancera-Pineda, J. E. Flowering patterns in three neotropical mangrove species: Evidence from a Caribbean island. *Aquatic Botany* **94**, 177-182 (2011).

14 Vandegehuchte, M. W. *et al.* Long-term versus daily stem diameter variation in co-occurring mangrove species: Environmental versus ecophysiological drivers. *Agricultural and Forest Meteorology* **192**, 51-58 (2014).

15 Aspinwall, M. J. *et al.* Salinity has little effect on photosynthetic and respiratory responses to seasonal temperature changes in black mangrove (Avicennia germinans) seedlings. *Tree Physiology* **41**, 103-118 (2021).

16 Vilas, M. P. *et al.* Night and day: Shrinking and swelling of stems of diverse mangrove species growing along environmental gradients. *PloS one* **14**, e0221950 (2019).

17 Sharma, S., Kamruzzaman, M., Hoque, A. R. & Hagihara, A. Leaf phenological traits and leaf longevity of three mangrove species (Rhizophoraceae) on Okinawa Island, Japan. *Journal of oceanography* **68**, 831-840 (2012).

18 Gnanamoorthy, P. *et al.* Seasonal variations of net ecosystem (CO2) exchange in the Indian tropical mangrove forest of Pichavaram. *Estuarine, Coastal and Shelf Science* **243**, 106828 (2020).

19 Muller, E., Lambs, L. & Fromard, F. Variations in water use by a mature mangrove of Avicennia germinans, French Guiana. *Annals of Forest Science* **66**, 803 (2009).

20 Ganguly, D., Ray, R., Majumder, N., Chowdhury, C. & Jana, T. K. Monsoonal Influence on Evapotranspiration of the Tropical Mangrove Forest in Northeast India. *American Journal of Climate Change* **2014** (2014).
